# Effects of the physicochemical properties of titanium dioxide nanoparticles, commonly used as sun protection agents, on microvascular endothelial cells

**DOI:** 10.1007/s11051-013-2130-3

**Published:** 2013-12-04

**Authors:** Claudia Strobel, Adriano A. Torrano, Rudolf Herrmann, Marcelina Malissek, Christoph Bräuchle, Armin Reller, Lennart Treuel, Ingrid Hilger

**Affiliations:** 1 Department of Experimental Radiology, Institute of Diagnostic and Interventional Radiology I, Jena University Hospital-Friedrich Schiller University Jena, Erlanger Allee 101, 07747 Jena, Germany; 2 Department of Chemistry and Center for NanoScience (CeNS), University of Munich (LMU), Butenandtstraße 5-13 (E), 81377 Munich, Germany; 3 Department of Physics, University of Augsburg, Universitaetsstraße 1, 86159 Augsburg, Germany; 4 Physical Chemistry, University of Duisburg-Essen, Universitaetsstraße 5-7, 45117 Essen, Germany; 5 Institute of Applied Physics and Center for Functional Nanostructures (CFN), Karlsruhe Institute of Technology (KIT), 76128 Karlsruhe, Germany; 6 Institut für Mikrotechnik Mainz GmbH, Carl-Zeiss-Str. 18-20, 55129 Mainz, Germany

**Keywords:** Endothelial cells, Environmental and health effects, Nanoparticle, Nanotoxicology, Sun protection agent, Titanium dioxide

## Abstract

Until now, the potential effects of titanium dioxide (TiO_2_) nanoparticles on endothelial cells are not well understood, despite their already wide usage. Therefore, the present work characterizes six TiO_2_ nanoparticle samples in the size range of 19 × 17 to 87 × 13 nm, which are commonly present in sun protection agents with respect to their physicochemical properties (size, shape, ζ-potential, agglomeration, sedimentation, surface coating, and surface area), their interactions with serum proteins and biological impact on human microvascular endothelial cells (relative cellular dehydrogenase activity, adenosine triphosphate content, and monocyte chemoattractant protein-1 release). We observed no association of nanoparticle morphology with the agglomeration and sedimentation behavior and no variations of the ζ-potential (−14 to −19 mV) in dependence on the surface coating. In general, the impact on endothelial cells was low and only detectable at concentrations of 100 μg/ml. Particles containing a rutile core and having rod-like shape had a stronger effect on cell metabolism than those with anatase core and elliptical shape (relative cellular dehydrogenase activity after 72 h: 60 vs. 90 %). Besides the morphology, the nanoparticle shell constitution was found to influence the metabolic activity of the cells. Upon cellular uptake, the nanoparticles were localized perinuclearly. Considering that in the in vivo situation endothelial cells would come in contact with considerably lower nanoparticle amounts than the lowest-observable adverse effects level (100 μg/ml), TiO_2_ nanoparticles can be considered as rather harmless to humans under the investigated conditions.

**Electronic supplementary material:**

The online version of this article (doi:10.1007/s11051-013-2130-3) contains supplementary material, which is available to authorized users.

## Introduction

Titanium dioxide (TiO_2_) nanoparticles are widely used in everyday items, like personal care products. One major application is the use as effective physical absorbers of UV rays in sun protection agents. However, there are some concerns in terms of risks to human health, because the cytotoxic potential of TiO_2_ nanoparticles is not well understood. In general, nanoparticles with diameters smaller than 100 nm are known to be more cytotoxic than larger particles (e.g., 3 μm in diameter) (Donaldson et al. 1998, 2001) as a result of the increased surface area (Oberdörster et al. 2005). In this context, it has been shown that erythrocytes treated with nano-sized TiO_2_ (20 nm) revealed abnormal sedimentation, hemagglutination, and hemolysis in contrast to their micro-sized counterparts (200 nm) (Li et al. 2008). Nowadays, a large number of different TiO_2_ nanoparticles are commercially available. They do not only differ in their crystalline structure (anatase and rutile), but also in their size, morphology, surface properties (like coating), agglomeration and sedimentation behavior (Bolis et al. 2012; Rampaul et al. 2007). All these factors play a crucial role for nanoparticle–cell interactions (Cho et al. 2010; Verma and Stellacci 2010). Anatase and rutile nanoparticles differ in the mobility of charge carriers, the width of the optical band gap and photoactivity (Mogyorosi et al. 2003; Ohno et al. 2001; Prieto-Mahaney et al. 2009). To date, there have been hardly any studies that elucidated systematically the impact of TiO_2_ nanoparticles on cells in dependence on particles’ properties. Although few studies have been addressing the effects of TiO_2_ nanoparticles on human vascular cells (Iavicoli et al. 2011), the majority of these studies deal with uncoated TiO_2_ nanoparticles, which are irrelevant for use in sun protection agents. Moreover, the particles were insufficiently characterized (e.g., no coating mentioned or no indication of TiO_2_ crystalline form). Such investigations are important from the view that a penetration of the nanoparticles through the skin and then to the blood vessel system could occur (Oberdörster et al. 2005) in areas of injured skin (wounds, lesions, and skin disease) or areas of skin flexing (Tinkle et al. 2003). Another way of nanoparticle access to the blood system is after the application of spray-on sun protection agents (Boxall et al. 2007) via inhalation (Nemmar et al. 2001, 2002). The subsequent interactions of nanoparticles with endothelial cells are thought to disturb endothelial cell activity, which is critically for wound healing, inflammation, and blood circulation and finally increase the risk of cardiovascular diseases (Zhu et al. 2011).

Thus, the aim of this study was to characterize the effects of TiO_2_ nanoparticle formulations on human microvascular endothelial cells (HMEC-1). To evaluate the effects of crystal structure, both anatase as well as rutile TiO_2_ nanoparticles were included into the study. In addition, the impact of TiO_2_ nanoparticles after being tagged with components of sun protection agents was studied by the use of corresponding nanoparticles isolated by extraction from them. To assess the impact on the metabolism of cells, the relative cellular dehydrogenase activity and the relative adenosine triphosphate (ATP) content were determined. The release of monocyte chemoattractant protein-1 (MCP-1) was used as marker for the elucidation of the pro-inflammatory impact. The data were analyzed in relation to size, surface coating, ζ-potential, agglomeration state, and deposition behavior of the nanoparticles.

## Methods and materials

### TiO_2_ nanoparticles used in this study

**Fig. 1:**
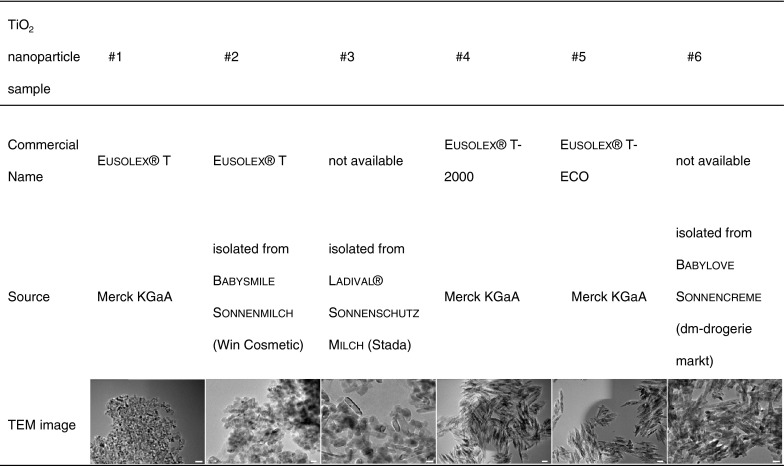
Overview of the used TiO_2_ nanoparticles. *Scale bar* 20 nm

Nanoparticle samples #1, #4, and #5 (Fig. 1) were obtained from Merck KGaA (Germany) as polydimethylsiloxane-coated TiO_2_ nanoparticles (Eusolex ^®^ T [sample #1]: anatase; Eusolex ^®^ T-2000 [sample #4] and Eusolex ^®^ T-ECO [sample #5]: rutile, additionally alumina as coating). These particles are used as raw materials for sun protection agents. In addition, TiO_2_ nanoparticles (samples #2, #3, and #6; Fig. 1) were directly isolated (see below) from sun protection agents with a protection factor of 50 + [Babysmile Sonnenmilch (Win Cosmetic, Germany), Ladival ^®^ Sonnenschutz Milch (Stada, Germany) and Babylove Sonnencreme (dm-drogerie markt, Germany)]. The properties of the TiO_2_ nanoparticles used in this study are summarized in Table 1.

**Table 1:** TiO_2_ nanoparticle characteristics

TiO_2_ nanoparticles	#1	#2	#3	#4	#5	#6
Crystalline polymorphic form of TiO_2_ (modification)	Anatase	Anatase	Rutile	Rutile	Rutile	Rutile
Size by TEM (nm)	19 ± 5 × 17 ± 4	19 ± 5 × 17 ± 4	64 ± 25 × 22 ± 5	87 ± 31 × 13 ± 3	87 ± 31 × 13 ± 3	48 ± 25 × 11 ± 5
Aspect Ratio	1.12	1.12	2.91	6.69	6.69	4.36
Size in H_2_O by DLS (nm)	1179 ± 309	68 ± 24	166 ± 49	113 ± 31	369 ± 65	3628 ± 746
Size in 0.2 % FBS medium by DLS (nm)	179 ± 34	157 ± 42	114 ± 19	184 ± 40	289 ± 106	278 ± 81
Size in 10 % FBS medium by DLS (nm)	97 ± 18	123 ± 71	2623 ± 1129	190 ± 47	56 ± 21	194 ± 50
SSA accessible to N_2_ molecules (m^2^/g) as obtained by BET measurements	50.0	2.8	22.0	83.2	83.8	42.0
ζ-Potential (mV) in 10 % FBS medium^a^	−15.0	−16.6	−14.9	−14.2	−13.7	−15.7
Schematic representation of nanoparticles with coating/secondary shell						
			

^a^ζ-potential in H_2_O and cell medium supplemented with 0.2 % FBS are presented in Table S1

To extract the TiO_2_ nanoparticles, the corresponding sun protection agent (10.0 g) was stirred vigorously with isopropanol (100 ml; Merck KGaA, Germany) during 2 h, sonicated for 10 min, and stirred again for 6 h. After filtering (G4 glass filter), the solid was dispersed again in isopropanol (100 ml), stirred, sonicated, and filtered. The treatment was repeated once again using 50 ml of isopropanol. In order to remove water-soluble ingredients and zinc oxide, 5 ml of 6 % HCl (v/v; Merck KGaA, Germany) was added to the residue (200 mg), the mixture was dispersed by sonication and left standing for 16 h. After centrifugation (4,300×*g*, 10 min), the pellet was washed with water (5 ml) and dried at 110 °C for 3 h. The total yield of nanoparticles was 210 mg (Ladival ^®^), 590 mg (Babysmile), and 1,550 mg (Babylove).

Particularly to study the internalization of TiO_2_ nanoparticles by cells it was necessary to label the particles with a fluorescence marker. The fluorescence dye *N*-(2,5-bis(dimethylethyl)phenyl)-*N*′-(3-(triethoxysilyl)-propyl-perylene-3,4,9,10-tetracarboxylic acid diimide (MPD) was prepared as already described (Blechinger et al. 2010). 25 mg of nanoparticles [Eusolex ^®^ nanoparticles (samples #1, #4 and #5, Fig. 1) or nanoparticles isolated from sun protection agents (samples #2, #3 and #6, Fig. 1)] were dispersed in ethanol (2.0 ml; Merck KGaA, Germany, Emsure quality), and MPD was added (0.06 mg). The mixture was stirred in a closed screw-cap glass vial at 145–150 °C (oil bath temperature) for 12 h. After cooling, the labeled nanoparticles were separated by at least three repetitions of centrifugation (4,300×*g*, 15 min) and redispersion in 1.0 ml ethanol (sonication) steps. Ethanol as solvent was replaced by Millipore water by three repetitions of centrifugation/redispersion (1.0 ml water) steps. The nanoparticles were air-dried. Fluorescence spectra were measured with a F900 luminescence spectrometer (Edinburgh Analytical Instruments, UK) using dispersions of the labeled nanoparticles in ethanol (excitation at 488 nm). The TiO_2_ nanoparticles were finally sterilized by autoclaving (121 °C, 20 min). Prior to each experimental step, the nanoparticle stock suspensions were vortexed and placed in an ultrasound bath (Bandelin Sonorex RK 52 H, Bandelin electronic GmbH & Co., KG, Germany; HF-power: 60 W_eff_) for 10 min for optimal redispersion.

### Nanoparticle characterization

#### Fourier transform infrared (FT-IR) measurements to determine the coating and secondary shell of TiO_2_ nanoparticles

To elucidate the coating composition and the presence and composition of the secondary shell on the TiO_2_ nanoparticles, FT-IR measurements were done with a Bruker Equinox 55 spectrometer in the range 400–4,000 cm^−1^, using the attenuated total reflectance (ATR) technique which does not require sample preparation (32 scans).

#### Transmission electron microscopy (TEM) to determine size and shape of TiO_2_ nanoparticles

To determine the nanoparticle size and shape, dispersions of the nanoparticles in ethanol were applied onto carbon-coated copper grids (Plano, Formvar/coal-film on a 200 mesh net). TEM pictures were obtained with a JEM 2011 (JEOL, Tokyo, Japan) instrument.

#### Particle size distribution and ζ-potential determinations

Dynamic light scattering (DLS) measurements were employed to determine the hydrodynamic diameter of the nanoparticles. Investigations on the electrophoretic mobility of the nanoparticles were carried out to determine the ζ-potential. Both studies were conducted with a Zetasizer equipment (Nano ZS Malvern Instruments, UK). Measurements were performed on colloidal nanoparticle suspensions either in Millipore water or cell culture media (see below). The concentration of nanoparticles for all samples was 100 μg/ml.

#### Determination of the specific surface area (SSA)

The SSA accessible to N_2_ molecules was obtained by Brunauer–Emmett–Teller (BET) measurements according to Brunauer et al. (1938). The measurements were conducted with a Quantachrome Nova 2000 system (Quantachrome GmbH & Co., KG, Germany).

#### Agglomeration and sedimentation studies

The agglomeration state and sedimentation behavior of the TiO_2_ nanoparticles were investigated via light microscopy especially prepared to mimic the exposure of cells with nanoparticles. The selected time points (3, 24, 48, and 72 h) for these investigations correspond to those applied for metabolic activity determination, and therefore they reveal the local concentration and agglomeration state of nanoparticles which approached the cells. Imaging was performed on a spinning disk confocal microscope based on Nikon Eclipse TE2000-E equipped with a Nikon Apo TIRF 100×/1.49 oil immersion objective. The differential interference contrast (DIC) mode was employed. In brief, 400 μL of the TiO_2_ colloidal suspensions (100 μg/ml) was added to one well of a Lab-tek^™^ chambered cover glass system. The bottom of the well was imaged at 3, 24, 48, and 72 h thereafter.

### Interactions between TiO_2_ nanoparticles and human serum albumin

To investigate the nanoparticle–protein interactions, circular dichroism (CD) measurements were carried out. Water was added to 10 μl of an 11.0 mg/ml human serum albumin (HSA) solution (in loBind^®^ tubes) to a total volume of 1,900 μl. 2 mg of each type of TiO_2_ nanoparticles (samples #1 to #6; Fig. 1) was dissolved in Millipore water (1 ml) and sonicated for 10 min. Thereafter, 100 μl of the 2 mg/ml nanoparticle stock-solution was added to the protein solutions, resulting in an overall nanoparticle concentration of 100 μg/ml in the measured solutions. All samples were incubated for 3 and 24 h at room temperature. CD measurements were carried out in the near UV region between 260 and 180 nm using an AVIV 62 A DS CD-spectrometer (slit width 5 μm; scanning step size 1 nm). Secondary structural elements were determined using the CD neuronal network (CDNN) software (Böhm et al. 1992).

### Cell culture experiments

The immortalized human microvascular endothelial cells (HMEC-1; Centers for Disease Control and Prevention, USA) were cultivated in Gibco^®^ MCDB 131 medium supplemented with 10 % (v/v) fetal bovine serum (FBS), 1 % (v/v) GlutaMAX^™^ I (100X; Life Technologies GmbH, Germany), 1 μg/ml hydrocortisone (Sigma-Aldrich Chemie GmbH, Germany), 10 ng/ml epidermal growth factor (Life Technologies GmbH, Germany) at 37 °C in a 5 % CO_2_ humidified environment. The growth medium was replaced every 2–3 days. Cells were passaged using Gibco^®^ trypsin (Life Technologies GmbH, Germany) until 70–85 % confluency. The cells were negatively for mycoplasma as routinely determined via PCR. For experimentation, cells were seeded at a density of 12,000 cells/cm^2^, allowed to attach for 24 h, and incubated with the nanoparticles (see below).

### Cellular uptake and intracellular localization of TiO_2_ nanoparticles

Uptake and intracellular localization of the TiO_2_ nanoparticles were determined semi-qualitatively via LSM imaging. Hereto, the cells were incubated with a nanoparticle concentration of 100 μg/ml. At 3, 24, 48, or 72 h later, the cells were washed with Hank`s BSS (PAA Laboratories GmbH, Austria), fixed with 3.7 % (v/v) formaldehyde (Carl Roth GmbH & CO., KG, Germany) in Hank`s BSS for 10 min at 4 °C, and washed again with cold Hank`s BSS. The cells were permeabilized with 0.1 % Triton X-100 (Sigma-Aldrich Chemie GmbH, Germany) in Hank`s BSS for 3 min and washed with Hank`s BSS. Subsequently, cellular F-actin was stained with Alexa-Fluor^®^-546 Phalloidin (5 units/ml; 20 min at room temperature; Life Technologies GmbH, Germany). Fluorescence-labeled cells were covered with Permafluor (Thermo Fisher, USA) containing Hoechst 33258 (0.2 μg/ml; AppliChem GmbH, Germany) to label cell nuclei. Finally, the cells were analyzed via confocal laser scanning microscopy (LSM 510, Carl Zeiss, Germany; filters: BP 560–615 for Alexa-Fluor^®^-546 Phalloidin; BP 420–480 for Hoechst, BP 505–550 for labeled nanoparticles; magnification: 40×).

### Assessment of metabolic activity of endothelial cells after nanoparticle treatment

#### Determination of relative cellular dehydrogenase activity

To determine the relative cellular dehydrogenase activity, HMEC-1 were treated with TiO_2_ nanoparticles at different concentrations (10 fg/ml, 100 fg/ml, 100 pg/ml, 100 ng/ml, and 100 μg/ml). After defined incubation times (3, 24, 48, and 72 h), the cells were washed with Hank`s BSS, and incubated with 20 μl/well Cell titer 96 Aqueous One Solution Reagent (Promega GmbH, Germany) in culture medium. Then, the absorbance of the supernatants containing the bioreduced MTS (formazan) was measured at 492 nm using a microplate reader (Sunrise^™^, Tecan Group Ltd., Switzerland). Data were presented as relative values normalized to nontreated control cell populations.

On the basis of these results, we determined the lowest-observable adverse effects level (100 μg/ml) and use this concentration for the further in vitro experiments.

#### Determination of relative cellular ATP level

To assess the relative cellular ATP content, cells were seeded (see above) and the culture medium was replaced with a fresh one containing 100 μg/ml TiO_2_ nanoparticles. Then, the cells were washed with Hank`s BSS at 3, 24, 48, and 72 h later and the CellTiter-Glo^®^ Luminescent Cell Viability Assay (Promega GmbH, Germany) was performed according to manufacturer’s instructions. The relative ATP content of the cells was calculated from the measured luminescence (LUMIStar Galaxy, BMG LABTECH GmbH, Germany) and expressed as relative values compared to untreated control cells.

To interpret the impact of the nanoparticles on endothelial cells, we considered the threshold for cytotoxicity according to DIN EN ISO 10993-5:2009-10.

### Assessment of MCP-1 release as marker of pro-inflammatory impact of TiO_2_ nanoparticles

To determine the pro-inflammatory impact of the nanoparticles, HMEC-1 were exposed to the different TiO_2_ nanoparticle formulations depicted in Fig. 1 (c = 100 μg/ml). To control the ability of HMEC-1 to produce MCP-1 after a corresponding stimulus, the cells were incubated with interleukin-1β (IL-1β; *c* = 2,000 pg/ml; Sigma-Aldrich Chemie GmbH, Germany) as positive control. The nanoparticles and the IL-1β were diluted in Gibco^®^ MCDB 131 medium supplemented with 0.2 % FBS. After 24, 48, and 72 h of incubation, the cell culture supernatants were collected. The MCP-1 content in the supernatant was determined using a commercial Human MCP-1 ELISA Kit (RayBiotech, USA) according to manufacturer’s instructions. In brief, 2.5 h after adding various MCP-1 standard dilutions and samples into appropriate wells of an anti-human MCP-1 coated microplate, the wells were washed and biotinylated antibody was added. After 1 h of incubation and washing steps, incubation of 45 min with horseradish peroxidase-conjugated streptavidin followed. After another washing step and an incubation of 30 min with 3,3′,5,5′-tetramethylbenzidine (TMB), sulfuric acid was added as stop solution and the absorbance were measured at 450 nm using a microplate reader (Sunrise^™^, Tecan Group Ltd., Switzerland). Samples were run in triplicate.

### Statistical analysis

The statistical analysis was carried out using IBM SPSS Statistics, version 19.0 (©2010 SPSS Statistics 19 Inc, an IBM Company, USA). Results were stated as means with standard deviation and considered as statistically different at *P* ≤ 0.05. Data were analyzed using ANOVA. The post hoc Bonferroni test was employed to determine differences between different treatment groups.

## Results

### The physicochemical properties of TiO_2_ nanoparticles

The TiO_2_ nanoparticles used in the present study are summarized in Fig. 1. They revealed distinct physicochemical properties. The shape of the anatase particles can be approximated as prolate ellipsoid (egg-shaped, Fig. 1), having a medium aspect ratio in the TEM pictures of 1.12 (core diameters: 19 × 17 nm; samples #1, #2; Fig. 1; Table 1), while rutile particles are rod-like with an aspect ratio up to 6.69 (core diameters: 87 × 13 nm; samples #4, #5; Fig. 1; Table 1). The hydrodynamic diameter of nanoparticles in Millipore water ranged from 68 nm (sample #2) to 3628 nm (sample #6). In presence of serum proteins [0.2 or 10 % FBS (v/v)], there was a distinct decrease of the hydrodynamic diameter detectable for nanoparticle sample #1, sample #5, and sample #6. In contrast, sample #2 and sample #3 revealed distinct increase whereas sample #4 showed only a trend toward an increase of hydrodynamic diameters compared to those measured in water (Table 1). If one compares the data from DLS measurements with TEM, distinct higher values were observed via DLS indicating nanoparticle agglomeration and aggregation. This effect was most prominent for sample #3 after incubation in serum-rich medium (10 % FBS). The mean diameter measured via DLS revealed agglomerates which were roughly 40 times larger than the individual nanoparticles measured via TEM. Only sample #5 showed comparable size distribution between DLS and TEM measurements in presence of serum proteins and consequently no clustering (Table 1).

The SSA for the unlabeled nanoparticles ranged between 2.8 and 83.8 m^2^/g (Table 1). The extracted sample #2 (2.8 m^2^/g) showed a very low SSA compared with the starting material (sample #1). Moreover the labeling process with a fluorescent dye increased the surface area accessible to N_2_ molecules in all cases (Table S2).

**Fig. 2:**
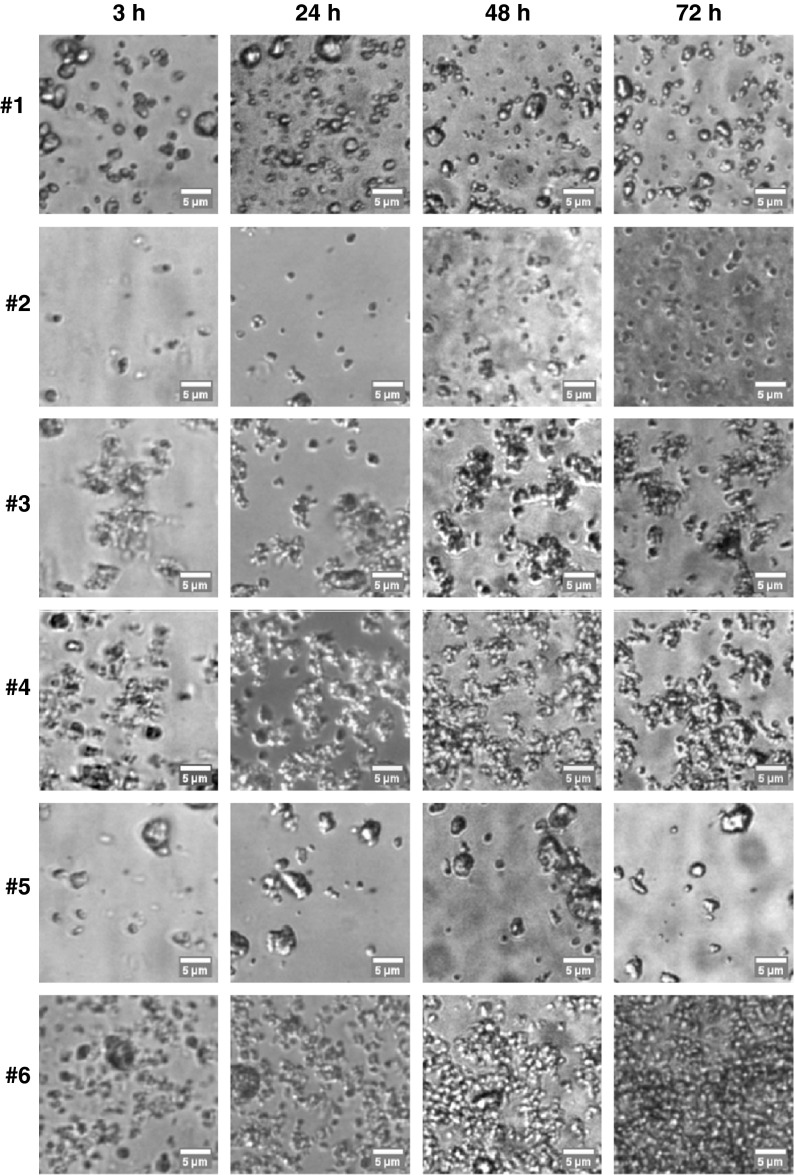
The investigated TiO_2_ nanoparticles differ enormously in their sedimentation behavior and agglomeration state. Colloidal suspensions were added to culture wells deprived of cells in order to mimic the exposure of nanoparticles to cells. Along the same time points, there were distinct differences in the amount of deposited nanoparticles and agglomeration state of different TiO_2_ nanoparticles. *Scale bar* 5 μm

Figure 2 presents representative images of the bottom of cell culture wells to reveal the agglomeration state and sedimentation behavior of the investigated TiO_2_ nanoparticles. Whereas the amount and size distribution of deposited sample #1, sample #4 and sample #5 nanoparticles did not change considerable between 3 and 72 h of incubation, the alterations for the three nanoparticle formulations isolated from sun protection agents (sample #2; sample #3; sample #6) were remarkable, resulting in a clear increase in the local amount of deposited nanoparticles with time. If the samples are compared among each other, for specific time points, the situation becomes even more complex. Taking for example the exposure of sample #1 after 24 h in comparison to the exposure of sample #2 TiO_2_ nanoparticles, one can see that considerably more sample #1 nanoparticles were deposited within the same incubation duration than sample #2 nanoparticles.

The ζ-potential of all nanoparticles in both cell media [serum-poor medium (0.2 % FBS) and serum-rich medium (10 % FBS)] ranged from −14 to −19 mV (pH 7; Table 1; Table S1). No correlation between size, shape, or surface coating of the nanoparticles was found.

The analysis of the composition of the nanoparticles by FT-IR spectroscopy confirmed the presence of dimethicone/simethicone coating in sample #2 and sample #3 (1,256 cm^−1^, symmetric bending of Si–CH_3_ groups, and slightly overlapping peaks at 1,016 and 1,096 cm^−1^ due to Si–O stretch and Si–O–Si bending) (Torrado et al. 1999). These bands were also present in the sample #1, sample #4, and sample #5. One could also detect residual materials from the formulations (secondary shell), which consisted of esters of aromatic acids (sample #2 and sample #3), and of polyols (sample #6). Sample #3 still contained a strong band at 1,060 cm^−1^ due to Si–O–Si units present in the surfactant stearalkonium hectorite.

The labeling of the nanoparticle with the fluorescence marker *N*-(2,5-bis(dimethylethyl)phenyl)-*N*′-(3-(triethoxysilyl)-propyl-perylene-3,4,9,10-tetracarboxylic acid diimide (MPD) did not alter the structural properties in terms of size, shape, or constitution of the secondary shell. Only the labeled variants showed fluorescence emission (dispersion in ethanol, excitation 488 nm led to emission at 540 and 580 nm, with a long tail from 600 to ca. 680 nm).

### Interactions between TiO_2_ nanoparticles and HSA

The presence of all different TiO_2_ nanoparticles led to changes in the secondary structure of the HSA molecules. In general, α-helical structure was lost and random structural elements became increasingly dominant (Table 2). As an exception from these observations, the nanoparticle samples #3 and #6 seemingly increased the relative fraction of α-helical structure in solution after 3 h, as compared to the native HSA. After 24 h, the α-helical structure in presence of these nanoparticles was found to be reduced. CD spectra were acquired for all nanoparticles and they did not show own CD signals in this wavelength region.

**Table 2:** α-Helix fraction, β-sheet, and random coil fraction of HSA in the presence of TiO_2_ nanoparticles after an incubation time of 3 and 24 h at room temperature

TiO_2_ nanoparticles	α-Helix (3 h) (%)	α-Helix (24 h) (%)	β-Sheet (3 h) (%)	β-Sheet (24 h) (%)	Random coil (3 h) (%)	Random coil (24 h) (%)
Serum albumin pure	68.8	68.8	4.5	4.5	11.9	11.9
#1	63.2	61.8	5.6	5.9	14.6	14.9
#2	64.5	33.0	5.4	18.1	14.8	31.3
#3	78.7	47.0	2.8	10.4	8.2	22.7
#4	53.1	33.3	8.2	17.9	21.2	31.1
#5	61.2	48.6	6.0	9.8	14.4	20.7
#6	80.1	30.0	2.5	20.6	7.6	33.9

### Metabolic impact of TiO_2_ nanoparticles on endothelial cells

**Fig. 3:**
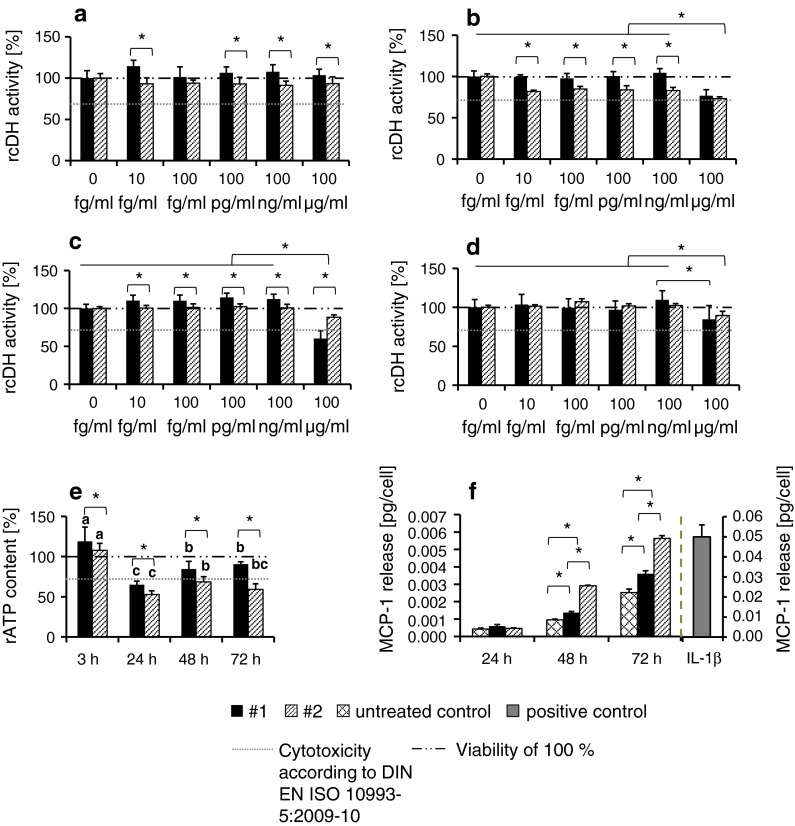
Impact of anatase TiO_2_ nanoparticles with different secondary shells on the metabolic activity of endothelial cells (HMEC-1). The graph represents the relative cellular dehydrogenase activity of cells, treated with sample #1 (without a secondary shell; *black bars*) and sample #2 (with a secondary shell; *stripped bars*) in dependence of different concentrations (10 fg/ml to 100 μg/ml) and incubation times (3 h (**a**), 24 h (**b**), 48 h (**c**), 72 h (**d**); *n* = 6 parallels) as well as the relative ATP content (**e** nanoparticle concentration: 100 μg/ml; *n* = 6 parallels). MCP-1 release after nanoparticle treatment (100 μg/ml) at different nanoparticle exposure times is shown (**f**; *n* = 3 parallels). Cells treated with IL-1β served as control of the ability of HMEC-1 to synthesize MCP-1 after stimulation. rcDH activity: relative cellular dehydrogenase activity; rATP content: relative ATP content; *a*, *b*, *c* indicate significant differences of the impact of one nanoparticle formulation between different exposure times, *P* ≤ 0.05; *asterisks* indicate significant differences between different nanoparticle formulations at a given time point, *P* ≤ 0.05

**Fig. 4:**
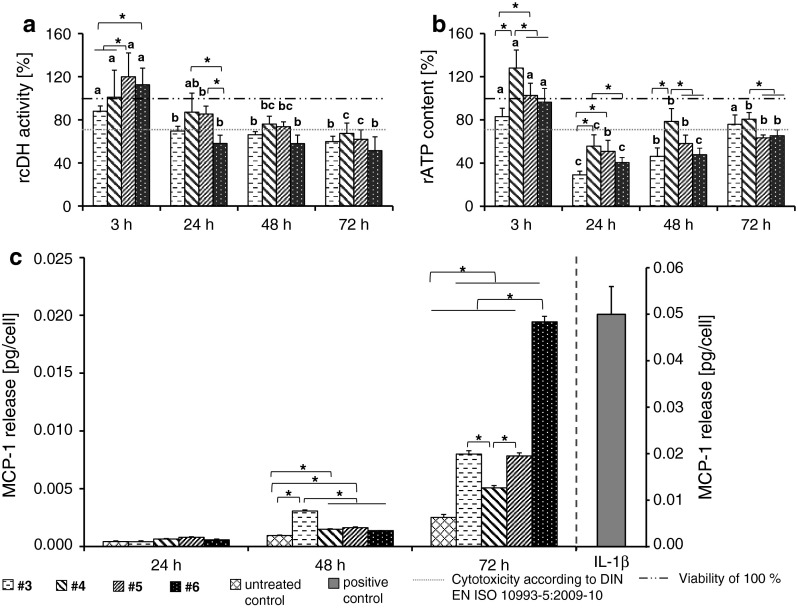
Effects on metabolic activity and pro-inflammatory impact of different rutile TiO_2_ nanoparticles (*c* = 100 μg/ml) are time and nanoparticle dependent. In general, a decrease of the relative cellular dehydrogenase activity (rcDH) was determined with increased exposure times of different rutile nanoparticles. Particularly, rutile nanoparticles with an alumina coating and a polyalcohol shell (sample #6) led after 24 h exposure to a strong decrease of cellular dehydrogenase activity [*n* = 6 parallels (**a**)]. The relative ATP (rATP) content was lowest after 24 h (*n* = 6 parallels (**b**)]. The MCP-1 release after nanoparticle treatment was highest for alumina-coated nanoparticles with a polyalcohol shell (sample #6) [*n* = 3 parallels (**c**)]. Cells treated with IL-1β served as control of the ability of HMEC-1 to synthesize MCP-1 after stimulation. rcDH activity: relative cellular dehydrogenase activity; rATP content: relative ATP content; *a*, *b*, *c* indicate significant differences of the impact of one nanoparticle formulation between different exposure times, *P* ≤ 0.05; *asterisks* indicate significant differences between different nanoparticle formulations at a given time point, *P* ≤ 0.05

Concentration-dependent analyses (from the fg/ml to the μg/ml range) revealed that all investigated TiO_2_ nanoparticles (anatase or rutile) had only a slight impact on the metabolic activity of endothelial cells (Fig. 3a–e for anatase nanoparticles; Fig. S1 [sample #4 as representative example for rutile nanoparticles]). Transient effects on the cellular dehydrogenase activity (with lower values than the threshold according to DIN EN ISO 10993-5:2009–10) were especially seen at a high concentration of 100 μg/ml (48 h of incubation, Fig. 3c; 24–72 h, Fig. 4a). Distinct correlations between size, aspect ratio, surface composition, agglomeration, sedimentation behavior, and metabolic activity were not observed (Tables S3, Table S4). Interestingly, the presence of an ester-based secondary shell correlates with the MCP-1 release of endothelial cells (Table S5).

### Impact of surface coating of TiO_2_ nanoparticles on the metabolic activity of endothelial cells

When analyzing the effect of anatase TiO_2_ nanoparticles (sample #1 and sample #2 in Fig. 1; Table 1) at a concentration of 100 μg/ml on the cells, a slight impact on the activity of cellular dehydrogenase together with a decrease of the relative ATP content (Fig. 3a–e) was observed. Yet, a remarkable increase of cellular MCP-1 release (48 and 72 h of incubation, Fig. 3f) in presence of the organic ester-based secondary shell compared to the bare nanoparticle counterpart was detected.

Similar relationships were encountered in relation to the rutile nanoparticles. Hereto, the commercially available nanoparticles containing a di/simethicone-alumina coating (sample #4; sample #5) revealed a gradual decrease in cellular dehydrogenase activity over time (Fig. 4a). In contrast, the nanoparticles isolated from sun protection agents (sample #3: di/simethicone coating with an ester secondary shell; sample #6: pure alumina coating with a polyol-based secondary shell) showed a rapid decrease of cellular dehydrogenase activity within 24 h of incubation, but for larger incubation times a further decrease was not detected (Fig. 4a). Moreover, the rutile TiO_2_ nanoparticles affect the cellular ATP content of endothelial cells (Fig. 4b). Particularly, sample #3 nanoparticles led to a strong decrease of ATP. In terms of the pro-inflammatory impact, rutile nanoparticles containing a polyalcohol-based secondary shell (sample #6) caused the strongest increase of MCP-1 release in comparison to the other investigated TiO_2_ nanoparticles (72 h of incubation). In contrast, nanoparticles with an ester-based secondary shell (sample #3) led to a higher MCP-1 release after 48 h of incubation compared with the other rutile nanoparticles (Fig. 4c and Table S5).

### The role of TiO_2_ nanoparticle crystal structure on the metabolic cell activity and pro-inflammatory response of endothelial cells

**Fig. 5:**
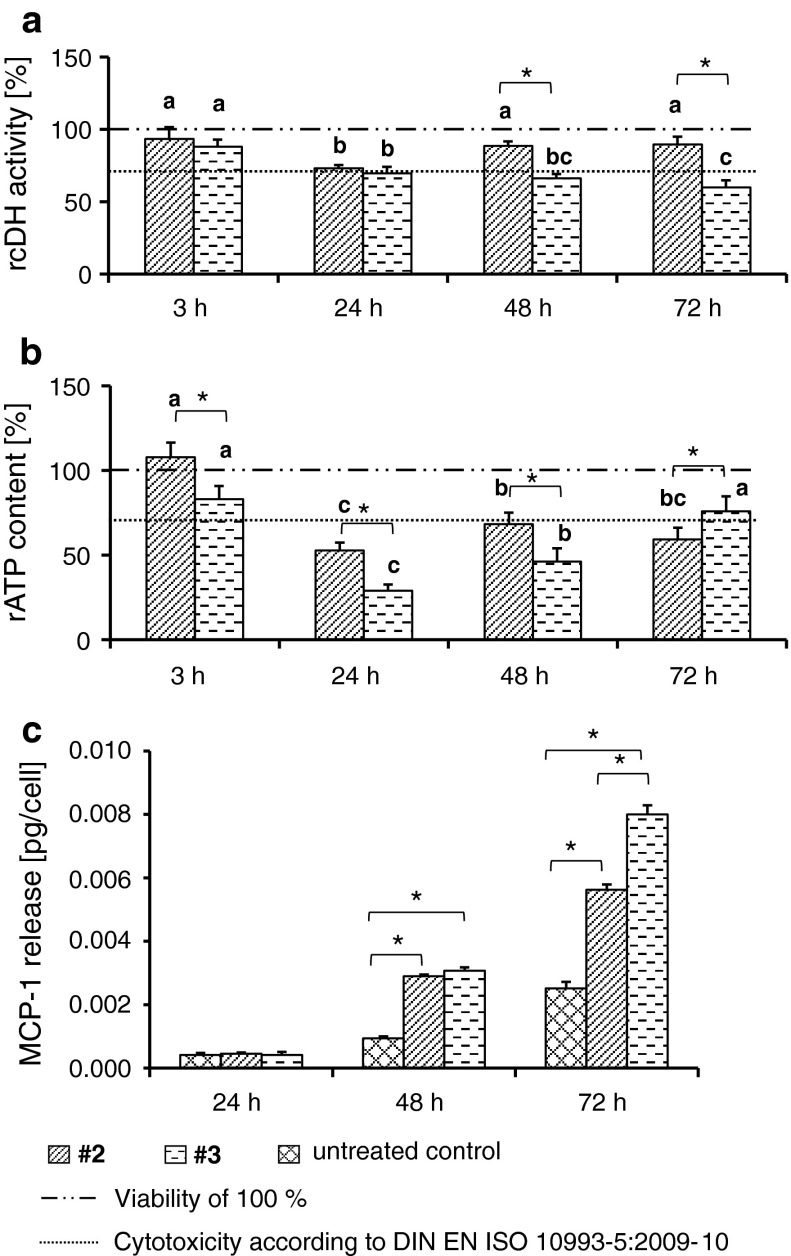
The role of TiO_2_ nanoparticle crystal structure (anatase, rutile; *c* = 100 μg/ml) on metabolic activity and pro-inflammation. Rutile TiO_2_ nanoparticles (sample #3) revealed after 48 and 72 h of exposure a stronger decrease of cellular dehydrogenase activity than their anatase counterparts [sample #2; *n* = 6 parallels (a)]. The relative ATP content was lowest after 24 h of exposure with both nanoparticle formulations. The ATP levels were different between rutile and anatase nanoparticles at given time points (*n* = 6 parallels (b)]. After 48 and 72 h nanoparticle treatment revealed an increase of MCP-1-release [*n* = 3 parallels (**c**)]. After 72 h of incubation rutile nanoparticles led to a stronger release of MCP-1. rcDH activity: relative cellular dehydrogenase activity; rATP content: relative ATP content; *a*, *b*, *c* indicate significant differences of the impact of one nanoparticle formulation between different exposure times, *P* ≤ 0.05; *asterisks* indicate significant differences between different nanoparticle formulations at a given time point, *P* ≤ 0.05

The comparison of slightly elliptical anatase (sample #2) and rod-like rutile (sample #3) nanoparticles containing comparable coating materials and aspect ratios revealed a higher impact of rutile nanoparticles on cellular dehydrogenase activity with increasing incubation times (Fig. 5a). At 24 and 48 h of incubation, the rutile nanoparticles also led to a stronger decrease of relative cellular ATP level (Fig. 5b) and a higher release of MCP-1 at time point 72 h of incubation (Fig. 5c).

### Intracellular localization of TiO_2_ nanoparticles

**Fig. 6:**
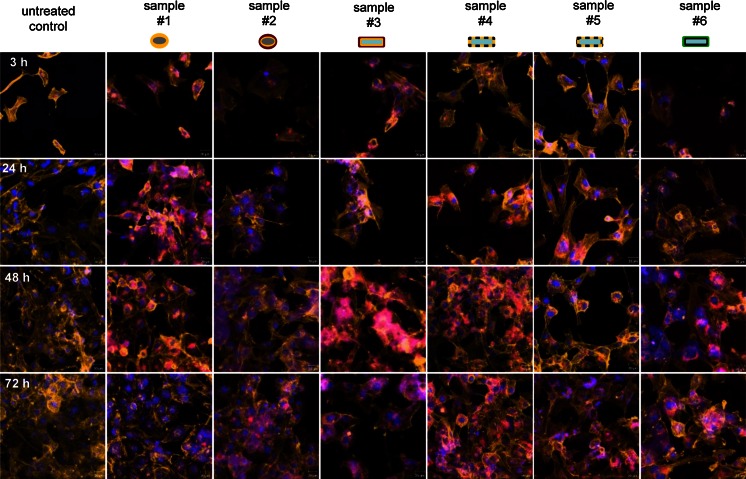
TiO_2_ nanoparticles are localized perinuclearly after cellular uptake. LSM pictures of HMEC-1 after different incubation times with different TiO_2_ nanoparticles (100 μg/ml). The nucleus (*blue*) was stained with Hoechst 33258 and the F-actin (*yellow*) with Alexa-Fluor^®^-546 Phalloidin. The TiO_2_ nanoparticles with MPD labeling are *red* in the pictures. The uptake was time dependent. Particularly, nanoparticles with a secondary shell consisting of esters revealed more diffuse structures of accumulation. Description of the schematic representation of nanoparticles is found in Table 1. MPD: *N*-(2,5-bis(dimethylethyl)phenyl)-*N*′-(3-(triethoxysilyl)propyl)-perylene-3,4,9,10-tetracarboxylic acid diimide; *scale bar* 20 μm

Nanoparticle uptake was time dependent and, from the morphological point of view, the nanoparticles were generally found to be localized perinuclearly (Fig. 6). Native nanoparticle formulations without a secondary shell (samples #1, sample #4, sample #5) as well as one nanoparticle sample with a polyalcohol-based secondary shell after isolation from sun protection agents (sample #6) revealed a focal intracellular accumulation. Nanoparticle formulations with an ester-based secondary shell (samples #2 and #3) showed rather diffuse features of accumulation (Fig. 6).

### Impact of the MPD fluorescence marker nanoparticle labeling on the metabolic activity of endothelial cells

Overall, the results obtained for labeled nanoparticles regarding cellular dehydrogenase activity (Fig. S2) and relative cellular ATP content (Fig. S3) were not considerably different to that of their unlabeled counterparts. Thus, labeling of nanoparticles with the fluorescence dye MPD has no considerable artificial effects on the metabolic activity of endothelial cells.

## Discussion

The present study demonstrates the impact of TiO_2_ nanoparticle formulations varying in their crystalline shape and surface coating on the metabolic activity of endothelial cells. In particular, the study yielded the following important results: (1) the TiO_2_ nanoparticles of the present study exhibited anatase or rutile crystal structure, (2) TiO_2_ nanoparticles isolated from sun protection agents displayed an organic surface coating containing esters or polyols, (3) the nanoparticle morphology was found not to be associated with the agglomeration and sedimentation behavior, (4) the different nanoparticle surface coatings did not modify the ζ-potentials distinctly, and (5) the nanoparticle impact on endothelial cells was rather low and only detectable at concentrations of 100 μg/ml. Moreover, distinct effects were found: (6) the rutile crystalline shape had an higher impact than the anatase-based one, the nature of the organic shell of rutile nanoparticles had an effect on the metabolic activity of the cells, and particularly the ester-based surface coating induced an increase in MCP-1, (7) nanoparticles were found to be localized perinuclearly with a tendency of varying accumulation patterns according to the physicochemical structure.

The observed surface areas of the investigated nanoparticles are well in the range of agglomerated TiO_2_ nanoparticles (Bolis et al. 2012). Assuming that any coating per se reduces the accessible surface area by clogging nanopores in the particles as well as the inter-particle volume, the increased SSA for the labeled nanoparticles can be attributed to a partial removal of the coating as a consequence to treatment with ethanol at 140–150 °C. The reason of the relatively low surface area of sample #3 might be due to the presence of the organic/inorganic surfactant stearalkonium hectorite in the formulation which is its most sticky part. The very low SSA of sample #2 is very probably due to some polymeric material with low solubility (VP/eicosene copolymer, acrylate/C_10–30_ alkyl acrylate crosspolymer, xanthan gum) present only in Babysmile sun protection agent that is responsible for this high degree of stickiness. Since the functional groups contained in such polymers (mainly esters) are the same as in many other ingredients of the formulations, IR spectroscopy does not allow determining their presence at the particle surface with certainty. In agreement with other publications (Bolis et al. 2012; Hsiao and Huang 2011; Sayes et al. 2006) it can be concluded that it is not the surface area obtained from BET measurements, which determines the biological behavior of coated TiO_2_ nanoparticles. In our case it is rather the chemical composition of the secondary shell.

All nanoparticles under investigation had negative ζ-potentials in the range of −14 to −19 mV. This is not sufficiently negative to prevent agglomeration induced by van der Waals forces (Jiang et al. 2009). In addition, the adsorption of proteins from the cell culture media has distinct effect on the agglomeration behavior of the nanoparticles, and this may result in a reduced agglomeration behavior by steric stabilization, or, on the contrary, it may lead to crosslinking of the nanoparticles (Allouni et al. 2009). Among the nanoparticles tested, those showing an ester-based secondary shell (sample #2; sample #3) revealed a comparable increased agglomeration in the presence of proteins (hydrodynamic diameter measured in Millipore water versus serum-rich medium; Table 1). All TiO_2_ nanoparticles under investigation affected the protein structure of albumin (the most abundant protein in blood plasma), which confirmed the adsorption of proteins. The increase in the relative fraction of α-helical structure in solution after 3 h by samples #3 and #6 compared to the native albumin and the subsequent reduced α-helical content of these nanoparticles after 24 h may well be caused by an induced chirality resulting from interactions between functional groups on the nanoparticle surface and in the protein. On longer timescales this may be outweighed by stronger structural changes in the protein. We note that the adsorption of proteins onto nanoparticles surfaces is a process essentially dependent on the nanoparticle surface chemistry and -area, among other factors (Abbas et al. 2010; Gebauer et al. 2012; Tenzer et al. 2011; Treuel and Nienhaus 2012).

Moreover, the agglomeration also influences the sedimentation of nanoparticles, which is an important parameter for in vitro experiments since the cells are exposed to nanoparticles under static conditions. Hereto, a higher sedimentation rate of nanoparticles led to a higher nanoparticle amount on the endothelial cell layer compared to nanoparticles with a lower rate of sedimentation, despite the fact that the cells were treated with a comparable nanoparticle concentration. For example, taking the exposure of sample #2 to cells after 24 h in comparison to sample #1 during the same period (Fig. 2), it is evident that the size and the number of nanoparticles in contact with cells would differ. Therefore, the obtained results for metabolic activity and pro-inflammation could be influenced by differences in agglomeration and sedimentation behavior of the nanoparticles rather than by the biological effects, a problem which has been poorly considered in nanocytotoxicological experiments up to now.

In general, our data show comparatively slight effects of TiO_2_ nanoparticles (anatase, rutile, aspect ratio up to ~7) on endothelial cells as opposed to other studies. For example, anatase or rutile nanoparticles (no surface coating mentioned) reduced the relative cellular dehydrogenase activity of mouse keratinocyte cells to 43.63 or 50.83 % (24 h exposure, 100 μg/ml), respectively (Braydich-Stolle et al. 2009). TiO_2_ nanoparticles (96 % anatase/4 % rutile; particle size <50 nm; no data on surface coating available) induced cell death in ~20 % and necrotic death in 60 % of all treated human umbilical vein endothelial cells (HUVEC, 24 h) (Montiel-Dávalos et al. 2012). Interestingly, no distinct impact was observed on the dehydrogenase activity of human dermal microvascular endothelial cells after exposure to 5 or 50 μg/ml TiO_2_ nanoparticles (70 nm, 72 h; no coating or aspect ratio mentioned) (Peters et al. 2004). It is known that TiO_2_ absorbs about 70 % of incident UV (Dunford et al. 1997). The produced single electrons are translocated to the nanoparticles surface, where they react with oxygen, hydroxyl ions or water to give superoxide and hydroxyl radicals, which initiate cellular oxidative stress (Dunford et al. 1997; Wolf et al. 2001). Coatings on the surface of TiO_2_ nanoparticles capture this radical formation. Therefore, the different effects between the mentioned studies and the present study may well be attributed to the presence of surface coatings on the nanoparticles in our study compared to uncoated TiO_2_ nanoparticles of other studies.

Remarkably, the anatase TiO_2_ nanoparticles did not alter the metabolic activity of human endothelial cells (cellular dehydrogenase and ATP level), independently of a secondary ester-based shell, whereas different effects were found in relation to the pro-inflammatory effect (sample #2). The underlying mechanisms remain unclear, and the findings basically point out the complexity on the diverse reactions of endothelial cells as a response to the exposure to TiO_2_ nanoparticles. Further studies should show to which extent organic components on the surface of the nanoparticles (e.g., after adsorbing organic components from sun protection agents) can trigger immunologic effects on endothelial cells in vivo.

In contrast, distinct surface-related effects were found for the rutile TiO_2_ nanoparticles. For example, the surface coating with the highest impact on dehydrogenase activity of endothelial cells was alumina (Al_2_O_3_) with the polyol secondary shell. Yet, pure Al_2_O_3_ nanoparticles (100 μg/ml; 24 h incubation; *d* = 40 nm (TEM), 267 nm (DLS); 36 mV) showed on human cardiac microvascular endothelial cells only slight effects on cellular dehydrogenase activity and pro-inflammatory response (Sun et al. 2011). This indicates that the observed effects of alumina-coated TiO_2_ nanoparticles (sample #6) are not mainly a result of the coating, but of the combination of TiO_2_ with Al_2_O_3_ and the resulting physicochemical properties.

When comparing the effects on cells in dependence on the surface coating, particularly the rutile TiO_2_ nanoparticles revealed distinct effects on metabolic activity (72 h of incubation) and a higher pro-inflammatory impact (MCP-1 release) when compared to their anatase counterparts. Assuming that degradation effects of dimethicone shells could occur in aqueous media (Auffan et al. 2010), it is conceivable that the higher pro-inflammatory impact of rutile nanoparticles could be associated with induced reactive oxygen species (ROS) that can particularly lead to activation of redox sensitive signaling pathways which result in transcription of pro-inflammatory cytokines and chemokines (Braydich-Stolle et al. 2009). The investigated rutile TiO_2_ nanoparticles (sample #3) could generate more ROS than the anatase counterparts (sample #2) due to their higher surface area (Table 1) (Jin et al. 2011). However, it can be assumed that partial dimethicone degradation and resulting ROS formation occurred only to a small extent, because overall the biological impact of the TiO_2_ nanoparticles was comparatively low.

The exposure of cells with TiO_2_ nanoparticles led to an increase of MCP-1 release compared to untreated cells, at least at comparatively high concentrations. MCP-1 is known to be an important chemo-attractant for the recruitment and activation of monocytes to the area of inflammation, and plays, among others, an important role in the development of chronic inflammation (Ikeda et al. 2002). Therefore, there is some evidence that TiO_2_ could have an impact on the induction of inflammation processes at least at high concentrations. Further investigations are necessary to elucidate the effects in the in vivo situation, e.g., under long-time exposure.

All TiO_2_ nanoparticles were found to be incorporated into the cells and located perinuclearly without any dependence on their corresponding crystal structure. A perinuclear localization of TiO_2_ nanoparticles was also found in relation to TiO_2_-based nanofilaments on H596 lung carcinoma cells (Magrez et al. 2009). In a recent investigation, rutile particles (*d* < 5 μm; without any coating) were located in the cytoplasm of HaCaT cells as small clusters or single particles, while anatase particles (*d* < 25 nm; without any coating) were found in the mitochondria and the nucleus (Jin et al. 2011). It can be assumed that the size of TiO_2_ nanoparticle agglomerates in the present study is too large for a translocation into nucleus or mitochondria. The uptake of nanoparticles was found to be time dependent. In this context, the comparatively low cellular ATP levels, which were observed particularly after 24 h of exposure, could be related to this process as an energy-consuming mechanism. Further research is needed to clarify this issue in more details. Since no differences were detected between fluorescence labeled and unlabeled TiO_2_ nanoparticles in relation to metabolic activity and physicochemical properties, the corresponding microscopy data of our study can be considered as highly accurate.

Even though distinct effects were observed, the principal point of our study is that TiO_2_ nanoparticles would be harmless at the investigated dose (lowest-observable adverse effects level at 100 μg/ml). For example, we consider that human beings after using an average of 30 g/d sun protection agent (equivalent to 1 mg/cm^2^ skin coverage) are exposed to a TiO_2_ nanoparticle amount of between 630 mg/d (sample #3) and 4,650 mg/d (e.g., our sample #6). If we assume that all nanoparticles on the skin would be translocated into the blood system (e.g., after skin injury), the blood TiO_2_ concentration could be between 126 and 930 μg/ml. This appears high, but the nanoparticle amount on endothelial cells per area would be between 0.0126 and 0.093 μg/cm^2^ in vivo compared to 29 μg/cm^2^ in vitro (equivalent to 100 μg/ml) as used in our study. Consequently, endothelial cells in vivo would come in contact with much lower nanoparticle amounts than those by which an effect was observed in cell culture experiments in vitro (100 μg/ml). Such concentrations rather overestimate the effects which could be found in vivo. Considering that TiO_2_ nanoparticles could be transmitted through the blood stream to fetuses during pregnancy, it cannot be excluded that their effects on the fetus would be different. However, with reference to the low concentrations that would reach the blood stream, these effects would likely be very low.

Furthermore, transmission of TiO_2_ from human wastes to other organisms for example plants and aquatic organisms is also possible. However, this was out of the scope of the present study and should be considered in future experimental setups.

## Conclusions

Taken together, coated TiO_2_ nanoparticles are expectedly harmless to endothelial cells. The observed effects on the cellular metabolic activity and pro-inflammatory response reveal a distinct dependence from the corresponding physicochemical nanoparticle properties, which are to be considered in a rather multiparametric way to better understand the underlying impact on living cells. Our study could contribute to present discussions on the potential effects of TiO_2_ nanoparticles from utilization as sun protection agents.

## Electronic supplementary material

Below is the link to the electronic supplementary material.

Supplementary material 1 (PDF 79 kb)
