# Supplementary material for: Effects of the physicochemical properties of titanium dioxide nanoparticles, commonly used as sun protection agents, on microvascular endothelial cells
Source: J Nanopart Res. 2013 Dec 4;16(1):2130. doi: 10.1007/s11051-013-2130-3 (PMC3890566; doi:10.1007/s11051-013-2130-3)
Supplement: Supplementary file 1 — Supplementary material 1 (PDF 79 kb) [file 11051_2013_2130_MOESM1_ESM.pdf]

Electronic Supplementary Material for Journal of Nanoparticle Research

Effects of the physicochemical properties of titanium dioxide nanoparticles, commonly used as sun protection agents, on microvascular endothelial cells

Claudia Strobel[1], Adriano A. Torrano[2], Rudolf Herrmann[3], Marcelina Malissek[4], Christoph Bräuchle[2], Armin Reller[3], Lennart Treuel[4,5], Ingrid Hilger[1§]

[1]Department of Experimental Radiology, Institute of Diagnostic and Interventional Radiology I, Jena University Hospital – Friedrich Schiller University Jena, Erlanger Allee 101, D-07747 Jena, Germany

[2]Department of Chemistry and Center for NanoScience (CeNS), University of Munich (LMU), Butenandtstraße 5-13 (E), D-81377 Munich, Germany

[3]Department of Physics, University of Augsburg, Universitaetsstraße 1, D-86159 Augsburg, Germany

[4]Physical Chemistry, University of Duisburg-Essen, Universitaetsstraße 5-7, D-45117 Essen, Germany

[5]Institute of Applied Physics and Center for Functional Nanostructures (CFN), Karlsruhe Institute of Technology (KIT), D-76128 Karlsruhe, Germany

§Corresponding author:

Prof. Dr. Ingrid Hilger, Institut für Diagnostische und Interventionelle Radiologie I, Universitätsklinikum Jena – Friedrich-Schiller-Universität Jena, Forschungszentrum Lobeda, Erlanger Allee 101, D-07747 Jena, Germany. Phone: 0049-3641-9325921, Fax: 0049-3641-9325922, e-mail: ingrid.hilger@med.uni-jena.de

Table S1 ζ-potential of TiO_2 nanoparticles

Sample	H_2O	Cell Medium 0.2% FBS	Cell Medium 10% FBS
#1	-17.8	-14.4	-15.0
#1 with MPD	-18.3	-14.8	-14.7
#2	-21.5	-14.9	-16.6
#2 with MPD	-18.3	-14.6	-15.6
#3	-25.1	-17.3	-14.9
#3 with MPD	-21.8	-17.3	-17.0
#4	-21.1	-15.8	-14.2
#4 with MPD	-17.8	-18.1	n.d.
#5	n.d.	-17.1	-13.7
#5 with MPD	-20.2	-14.9	-16.9
#6	-20.5	-14.4	-15.7
#6 with MPD	-16.6	-16.9	-19.4

MPD: *N*-(2,5-bis(di-methylethyl)phenyl)-*N'*-(3-(triethoxysilyl)-propyl)-perylene-3,4,9,10-tetracarboxylic acid diimide; n.d.: not determined.

Table S2 Specific surface area (SSA) of unlabeled and labeled TiO_2 nanoparticles accessible to N_2 molecules as obtained by BET measurements

Sample	SSA [m^2/g]
#1	50.0
#1 with MPD	76.8
#2	2.8
#2 with MPD	34.6
#3	22.0
#3 with MPD	32.7
#4	83.2
#4 with MPD	89.4
#5	83.8
#5 with MPD	93.0
#6	42.0
#6 with MPD	85.1

MPD: *N*-(2,5-bis(di-methylethyl)phenyl)-*N'*-(3-(triethoxysilyl)propyl)-perylene-3,4,9,10-tetracarboxylic acid diimide.

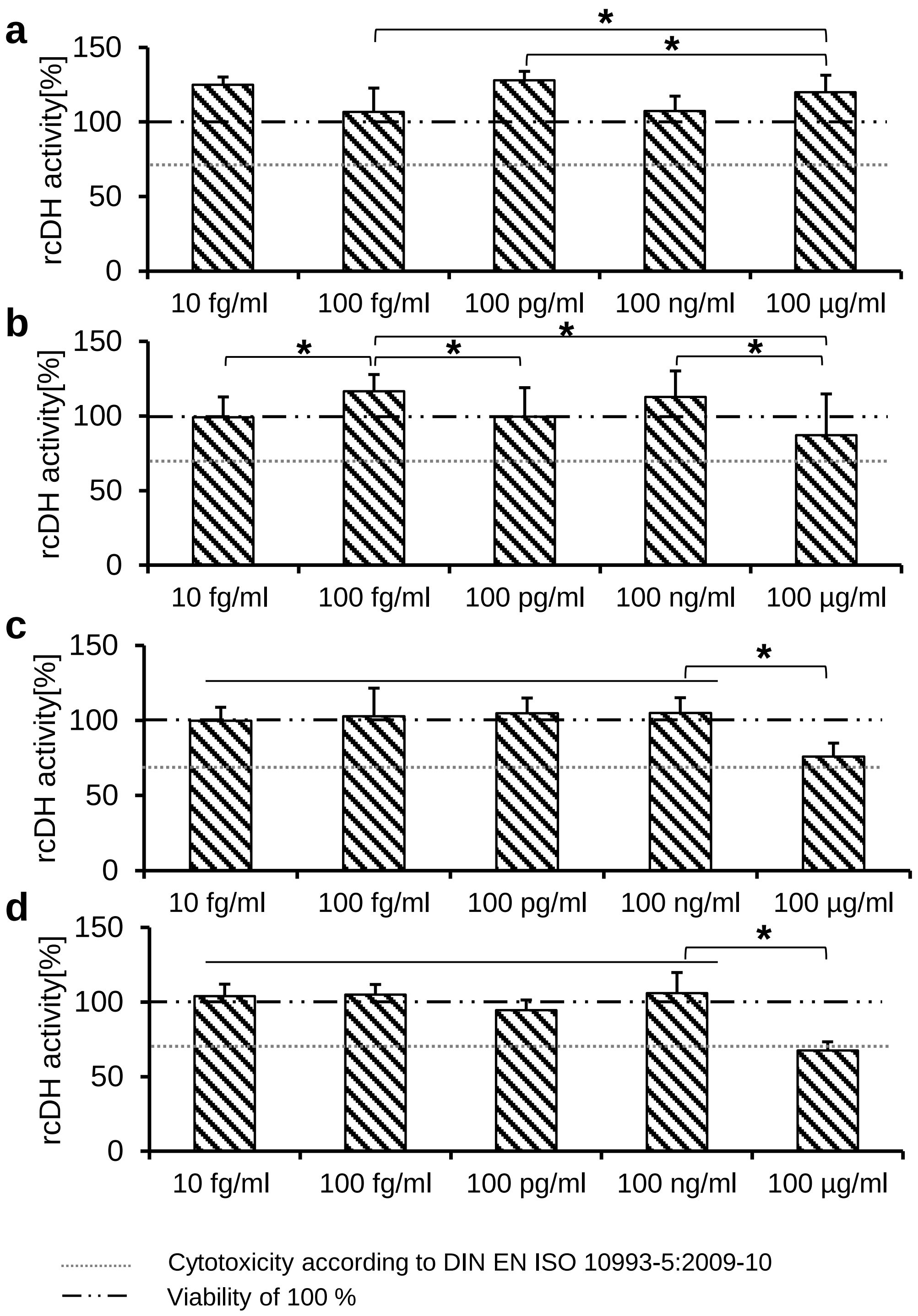

Fig. S1 Rutile TiO_2 nanoparticle sample #4 revealed considerable effects on cellular dehydrogenase activity at the highest concentration (100 µg/ml). Relative cellular dehydrogenase activity in dependence on the nanoparticle concentration (10 fg/ml to 100 µg/ml) and time (3 h (a); 24 h (b); 48 h (c); 72 h (d)). *asterisks indicate significant differences, $P \leq 0.05$; n = 6 parallels

Table S3 There was no correlation detectable between the effect on cellular dehydrogenase activity and the physicochemical properties of the nanoparticles

TiO_2 nano-particle sample	relative cellular dehydro-genase activity [%] [a]	crystalline polymorphic form of TiO_2	ζ-potential	Coating		Second. shell		Sedimen-tation (10% FBS)	Agglomeration (10% FBS)	mean Size (10% FBS)	Size [nm] by TEM	Aspect Ratio (TEM)
				si	al	e	hc					
#2	88.53	anatase	-16.6	x		x		*	**	123	19 x 17	1.12
#4	76.07	rutile	-14.2	x	x			**	**	190	87 x 13	6.69
#5	73.59	rutile	-13.7	x	x			*	*	56	87 x 13	6.69
#3	66.17	rutile	-14.9	x		x		**	***	2623	64 x 22	2.91
#1	60.18	anatase	-15.0	x				**	**	97	19 x 17	1.12
#6	58.08	rutile	-15.7		x		x	**	**	194	48 x 11	4.36

Data for time point 48 h after nanoparticle exposure; ordered from the highest to the lowest cellular dehydrogenase activity. [a] mean; x means "presented"; *slightly; **medium; ***strongly; si: simethicone; al: alumina; e: esters; hc: hydrophilic compounds; second.: secondary.

Table S4 There was no effect on the relative cellular ATP content in dependence on the physicochemical properties of the nanoparticles detectable

TiO_2 nano-particle sample	relative ATP content [%] [a]	crystalline polymorphic form of TiO_2	ζ-potential	Coating		Second. shell		Sedimen-tation (10% FBS)	Agglomeration (10% FBS)	mean Size (10% FBS)	Size [nm] by TEM	Aspect Ratio (TEM)
				si	al	e	hc					
#1	84.51	anatase	-15.0	x				**	**	97	19 x 17	1.12
#4	78.61	rutile	-14.2	x	x			**	**	190	87 x 13	6.69
#2	68.48	anatase	-16.6	x		x		*	**	123	19 x 17	1.12
#5	58.16	rutile	-13.7	x	x			*	*	56	87 x 13	6.69
#6	47.88	rutile	-15.7		x		x	**	**	194	48 x 11	4.36
#3	46.32	rutile	-14.9	x		x		**	***	2623	64 x 22	2.91

Data for time point 48 h after nanoparticle exposure; ordered from the highest to the lowest ATP content. [a] mean; x means "presented"; *slightly; **medium; ***strongly; si: simethicone; al: alumina; e: esters; hc: hydrophilic compounds; second.: secondary.

Table S5 The presence of an ester-based secondary shell correlates with the MCP-1 release of endothelial cells

TiO_2 nano-particle sample	MCP-1 [fg/cell] [a]	crystalline polymorphic form of TiO_2	ζ-potential	Coating		Second. shell		Sedimen-tation (0.2% FBS)	Agglomer-ation (0.2% FBS)	mean Size (0.2% FBS)	Size [nm] by TEM	Aspect Ratio (TEM)
				si	al	e	hc					
#3	3.07	rutile	-14.9	x		x		**	**	113.5	64 x 22	2.91
#2	2.91	anatase	-16.6	x		x		***	**	157.4	19 x 17	1.12
#5	1.63	rutile	-13.7	x	x			*	**	288.5	87 x 13	6.69
#4	1.49	rutile	-14.2	x	x			**	**	183.8	87 x 13	6.69
#6	1.38	rutile	-15.7		x		x	***	**	277.5	48 x 11	4.36
#1	1.37	anatase	-15.0	x				**	**	178.8	19 x 17	1.12

Data for time point 48 h after nanoparticle exposure; ordered from the highest to the lowest MCP-1 release. [a] mean; x means “presented”; *slightly; **medium; ***strongly; si: simethicone; al: alumina; e: esters; hc: hydrophilic compounds; second.: secondary.

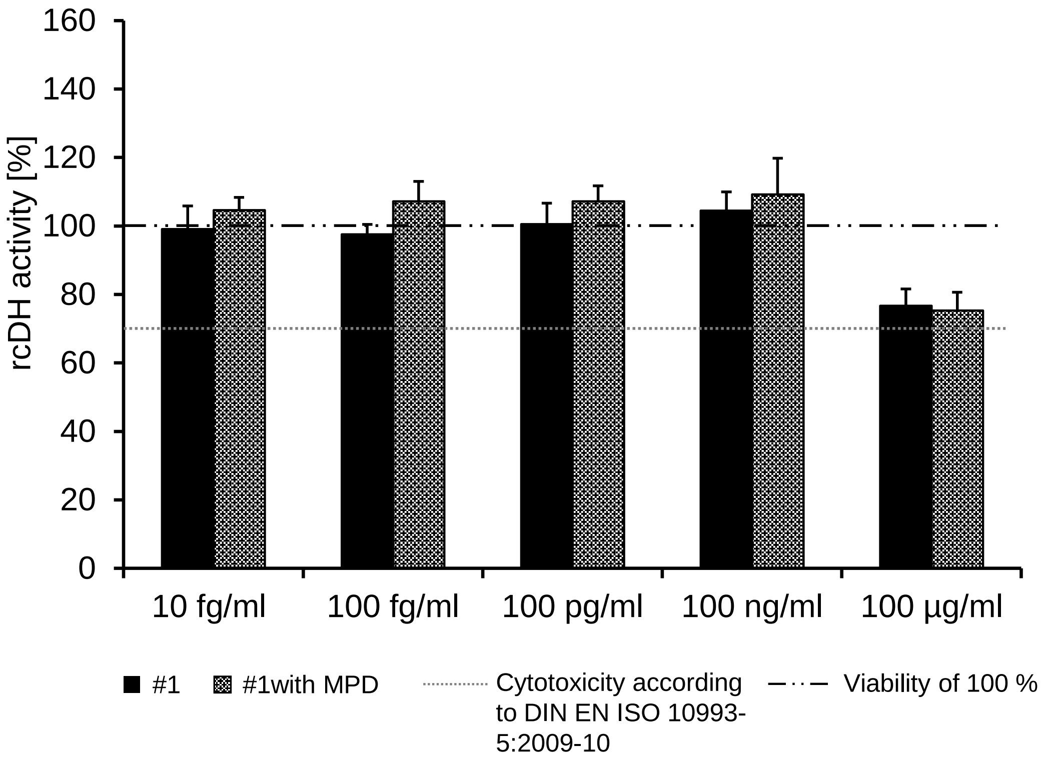

Fig. S2 The labeling of the coated TiO_2 nanoparticles with the perylene-derived fluorescence marker MPD had no artificial effect on cellular dehydrogenase activity of endothelial cells. The graph shows the relative dehydrogenase activity after 24 h incubation time for sample #1 nanoparticles with and without perylene label (black bars: sample #1; striped bar: sample #1with perylene label). rcDH activity: relative cellular dehydrogensae activity; MPD: *N*-(2,5-bis(dimethylethyl)phenyl)-*N*’-(3-(triethoxysilyl)propyl)-perylene-3,4,9,10-tetracarboxylic acid diimide; n = 6 parallels

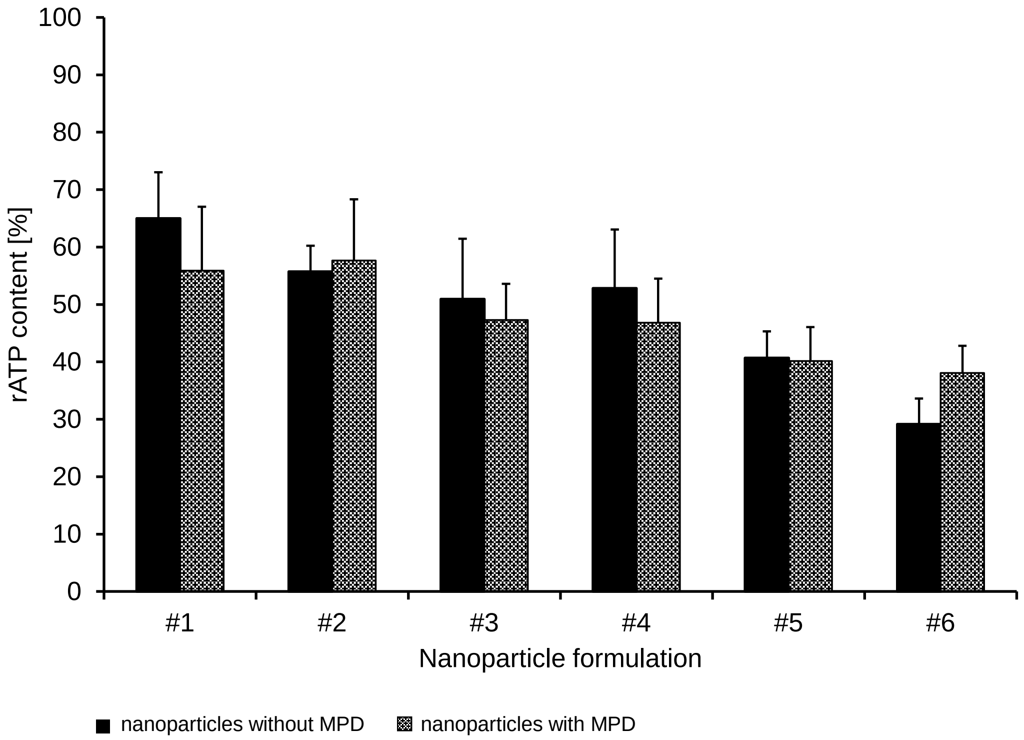

Fig. S3 The labeling of the coated TiO_2 nanoparticles with the perylene-derived fluorescence marker MPD had no considerable artificial effect on ATP content of endothelial cells. The graph shows the relative ATP level [%] after 24 h incubation time for TiO_2 nanoparticles (c = 100 µg/ml) without MPD (black bars) and with MPD (striped bar). rATP content: relative ATP content; MPD: *N*-(2,5-bis(dimethylethyl)phenyl)-*N*'-(3-(triethoxysilyl)-propyl)-perylene-3,4,9,10-tetracarboxylic acid diimide; n = 6 parallels
